# Climate adaptation and functional constraints drive pollen evolution in Apiales

**DOI:** 10.1111/nph.70824

**Published:** 2025-12-15

**Authors:** Jakub Baczyński, Krzysztof Spalik, John M. Burke, Łukasz Banasiak

**Affiliations:** ^1^ Institute of Evolutionary Biology, Faculty of Biology, Biological and Chemical Research Centre University of Warsaw Warsaw 02‐096 Poland; ^2^ Department of Plant Biology, Miller Plant Sciences University of Georgia Athens GA 30602 USA; ^3^ The Plant Center University of Georgia Athens GA 30602 USA

**Keywords:** Apiales, climate niche, evolution, harmomegathy, morphology, pollen

## Abstract

- Pollen grains exhibit remarkable morphological diversity, shaped by selective pressures from environmental factors and mechanical constraints. Here, we investigate macroevolutionary patterns of pollen morphology in Apiales, an order of angiosperms with significant ecological and geographical diversity, to disentangle the roles of climate and functional constraints.
- We analyzed pollen morphology in 158 species of Apiales using morphometric and multivariate evolutionary approaches to evaluate the influence of climate and biomechanical constraints on traits such as pollen wall thickness, aperture structure, and overall grain shape, and to test for evidence of harmomegathy‐related adaptation.
- Our results reveal three key findings. First, climate showed no significant effect on pollen size, challenging long‐standing assumptions. Second, climate strongly influences pollen architecture, with drier, more seasonal climates being consistently associated with reduced apertures and thicker pollen walls. Finally, we detected an evolutionary lag, with changes in pollen wall thickness preceding aperture modifications, indicating that biomechanical constraints have shaped evolutionary trajectories.
- These results demonstrate that climate‐driven adaptations in pollen architecture are mediated by functional constraints, consistent with a dynamic interaction between environmental selection and biomechanical properties of the pollen wall.

## Introduction

Pollen grains are the male gametophytes of seed plants, comprising a few cells enclosed by a protective layer known as the sporoderm (i.e. pollen wall). Despite considerable evolutionary reduction and a shared function across all spermatophytes, pollen displays remarkable variability in shape and size. The grains range from small, smooth, and spherical forms to highly elaborate ones, featuring spines, furrows, or unique ornamentation (Punt *et al*., 2007; Traverse, 2007). This morphological diversity is predominantly observed in the exine, the outer layer of the sporoderm, which consists of a complex of chemically inert biopolymers known as sporopollenin (Li *et al*., 2019). The exine's exceptional resistance contributes to a strong potential for pollen grains to fossilize (Campbell, 1991; Hesse *et al*., 1999), providing unparalleled opportunities to study the ecology and biogeography of past vegetation. Fossilized pollen has frequently been used as a source of calibration points for divergence time estimation across various groups of angiosperms (Soltis *et al*., 2002; Bell *et al*., 2005, 2010; Moore *et al*., 2007; Magallón, 2014; Magallón *et al*., 2015) whose origins and early diversification remain the subject of widespread debate (Sauquet & Magallón, 2018; Sauquet *et al*., 2022; Smith & Beaulieu, 2024).

The observed diversity in pollen grain shape and size reflects the influence of underlying developmental processes, as well as biotic (i.e. plant–pollinator interactions) and abiotic (i.e. climate) factors that shape them. Pollen development begins with the formation of microspores within the anther locules after meiosis of diploid microsporocytes. These haploid microspores are initially released as a tetrad before separating and undergoing vacuolization, followed by an asymmetric mitotic division to form a large vegetative cell and a smaller generative cell. In many angiosperms, the generative cell undergoes one further division to yield two sperm cells before anthesis. During maturation, the vegetative cell accumulates starches, lipids, and proteins, while the pollen wall is completed. The dehydration of the cytoplasm in the final stages of maturation arrests metabolic processes in pollen grains and prepares them for prolonged exposure to adverse conditions during their transfer from the anther to the stigma (McCormick, 1993; Borg *et al*., 2009; Twell, 2011). Upon reaching the stigma, pollen grains rehydrate, metabolic activity resumes, and swelling causes apertures – specialized regions of the sporoderm – to open, permitting pollen tube germination. To buffer the volumetric changes of dehydration and subsequent rehydration without structural failure, the sporoderm undergoes elastic folding and bending, a biomechanical process known as harmomegathy (Wodehouse, 1935; Payne, 1972). The survival of shed pollen grains thus depends on external environmental factors (Muller, 1979; Ejsmond *et al*., 2011; Franchi *et al*., 2011), harmomegathal efficiency, and a combination of internal physiological mechanisms (Gaff & Oliver, 2013) that contribute to its desiccation tolerance (Hoekstra & Bruinsma, 1979; Pacini, 1996; Nepi *et al*., 2001; Franchi *et al*., 2002, 2011; Pacini *et al*., 2006).

Despite the pivotal role of pollen grains in advancing our understanding of plant macroevolution, significant uncertainties persist regarding the primary drivers that have shaped their extant diversity. While many morphological characters are likely evolutionarily neutral (i.e. minute differences in exine structure), climate variation may exert selective pressure on specific traits such as aperture length or sporoderm thickness, which often evolve in concert with others due to biomechanical constraints. Comparative analyses across angiosperms indicate that pollen size tends to increase with temperature, whereas elongation (P/E ratio) is only weakly climate‐dependent (Ejsmond *et al*., 2011, 2015), and other studies highlight habitat moisture as a determinant of exine ornamentation, aperture number and length, and wall thickness (Lu *et al*., 2015; Luo *et al*., 2015; Fatmi *et al*., 2020). At the same time, biomechanical modeling underscores mechanical stress as a major selective force, showing that harmomegathal folding is facilitated by local weak spots – such as apertures or thin exine regions – that reduce the risk of irregular buckling (Katifori *et al*., 2010), with final folding patterns further influenced by elongation, wall elasticity, and ornamentation (Heslop‐Harrison, 1979; Payne, 1981; Matamoro‐Vidal *et al*., 2016; Božič & Šiber, 2020). The striking disparity in pollen morphology suggests that efficient changes in volume and shape during dehydration/rehydration can be achieved through a wide range of architectural solutions (Halbritter & Hesse, 2004; Volkova *et al*., 2013; Banks & Rudall, 2016). From this perspective, different morphologies represent ‘syndromes’ or adaptive peaks, in which multiple traits evolve in close coordination to preserve the structural integrity of the pollen wall. Thus, while climate may influence pollen diversity, its effects are intertwined with trade‐offs imposed by mechanical demands and shaped by phylogenetic inheritance.

In this study, we investigated the morphology of Apiales pollen and assessed the impact of climatic factors and potential harmomegathy‐related constraints on its evolution. Apiales are a relatively large angiosperm order comprising *c*. 5500 species (Kadereit & Bittrich, 2019), including well‐known members such as carrot (*Daucus carota* L.), common ivy (*Hedera helix* L.), and Japanese cheesewood (*Pittosporum tobira* (Thunb.) W.T. Aiton). Among the Apiales, early‐diverging families such as Pennantiaceae, Griseliniaceae, Torricelliaceae, Araliaceae, and Myodocarpaceae primarily consist of tropical and subtropical trees and shrubs (Nicolas & Plunkett, 2014). By contrast, the predominantly herbaceous Apiaceae underwent significant diversification in temperate regions of North America and Eurasia (Banasiak *et al*., 2013; Calviño *et al*., 2016), as well as in the Mediterranean region and across West and Central Asia.

**Fig. 1:**
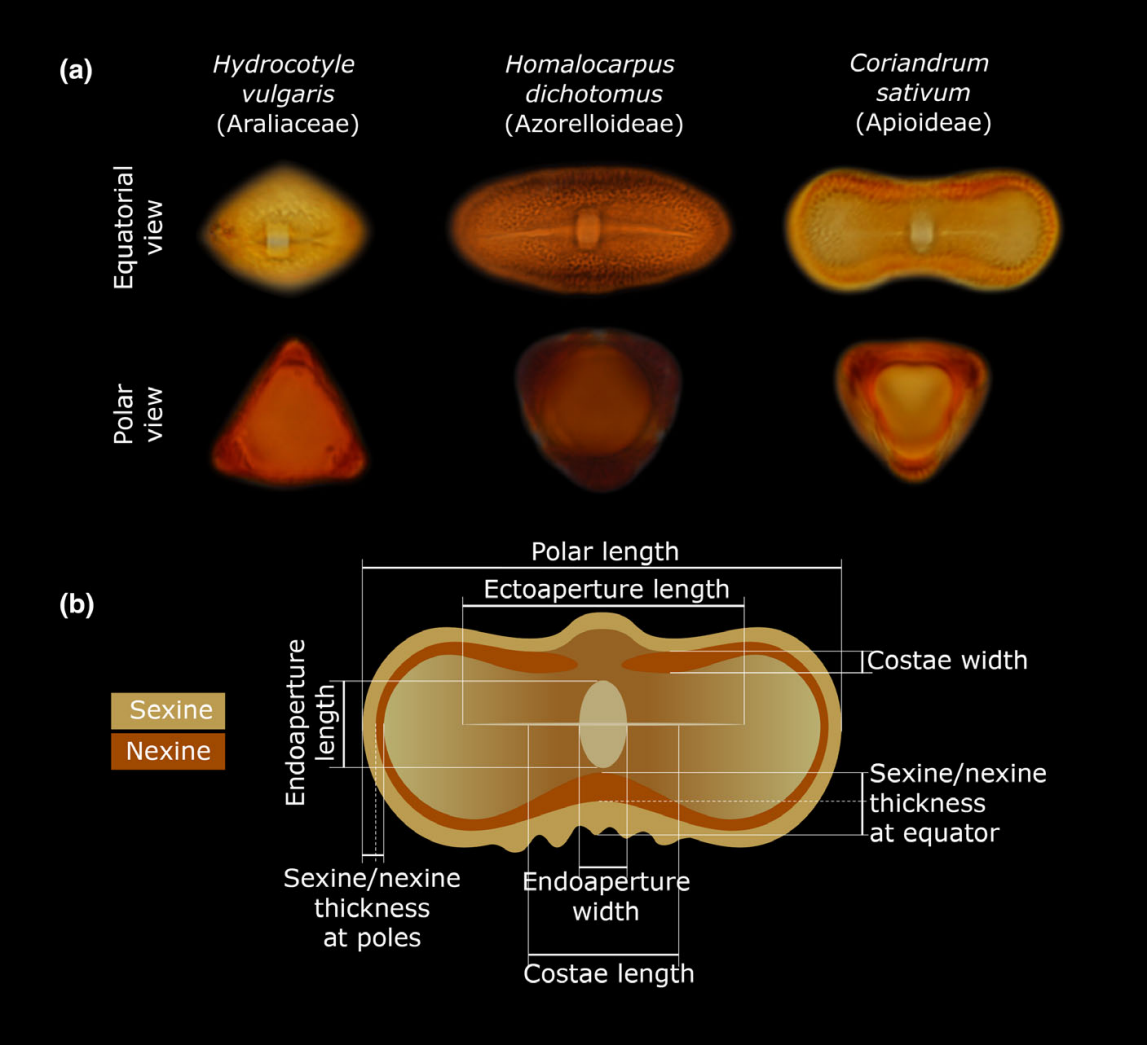
The diversity of pollen morphology in Apiales. (a) Early‐diverging tropical and subtropical representatives of the order (i.e. *Hydrocotyle vulgaris*, Araliaceae) typically possess relatively short pollen grains with well‐developed apertures and straight mesocolpia in the polar view; the apertures are situated at the corners of the triangular outline. In nonapioid umbellifers (i.e. *Homalocarpus dichotomus*), pollen is elongated and elliptical, with long ecto‐ and endoapertures, and generally exhibits a more circular shape in polar view. In Apioideae (i.e. *Coriandrum sativum*), both ecto‐ and endoapertures are reduced (sometimes to approximately half their original length), and the pollen is characteristically bone‐shaped, with sharply bent mesocolpia in polar view; in this case, apertures are located along the ‘sides’ of the triangle. (b) Schematic diagram illustrating the morphological traits measured in this study.

Pollen grains in Apiales generally exhibit a relatively uniform architecture. Exine ornamentation is typically striate in early‐diverging lineages, reticulate in Araliaceae and Myodocarpaceae, and predominantly striate or rugulate in Apiaceae. Pollen is almost universally trinucleate (Brewbaker, 1967), tricolporate, and lacks specialized dispersal structures (Chao, 1954; Ting *et al*., 1964; Tseng & Shoup, 1978; Tseng, 1980; Tseng *et al*., 1983; Henwood, 1991; Wen & Nowicke, 1999; Baczyński *et al*., 2021). However, there is a notable trend toward uneven sporoderm thickening and a shortening of compound apertures (Fig. 1a), particularly within Apiaceae (Cerceau‐Larrival, 1962, 1967; Baczyński *et al*., 2021). Herein, we demonstrate that while climate is not a strong predictor of pollen volume in Apiales, alterations in aperture structure and pollen wall thickness serve as signatures of past biogeographic shifts and adaptations to the drier and more seasonal climates of the Northern Hemisphere. The results from multivariate macroevolutionary model fitting and morphological investigation suggest that these changes in pollen structure caused a gradual shift in harmomegathal mechanisms from the apertures to other areas of the sporoderm. Taken together, these findings are consistent with theoretical predictions (Katifori *et al*., 2010; Božič & Šiber, 2020).

## Materials and Methods

### Morphological dataset

The analyses presented in this study are based on the dataset from Baczyński *et al*. (2021). In brief, the original study examined acetolyzed pollen from 417 herbarium specimens, representing 158 species and 127 genera, spanning all major lineages of Apiales (Supporting Information Fig. S1). Most species (91%) were represented by at least two specimens, preferably from different collections to minimize misidentification. To restore pollen to its natural shape, anthers isolated from dried specimens were rehydrated in water before acetolysis (Reitsma, 1969). To minimize further deformation during storage, slides were mounted in pure glycerin supported by a paraffin ring and analyzed within a few days of preparation. For each specimen, 10 representative pollen grains were measured – five in side view and five in colpus view.

In this work, our focus was on 10 quantitative traits (Fig. 1b) that encompassed aspects such as grain size, wall stratification and thickness, as well as aperture morphology: polar length (P), ectoaperture length relative to polar length (ECT/P), endoaperture length relative to polar length (ENDL/P), endoaperture elongation (ENDL/ENDW), wall thickness at the equator relative to polar length (WE/P), wall thickness at poles relative to polar length (WP/P), costae width relative to nexine at the poles (COSW/NP), costae length relative to polar length (COSL/P), and relative nexine thickness at the equator (NE/WE) and at the poles (NP/WP).

Pollen grain shape was analyzed using the methodology outlined by Kriebel *et al*. (2017). Briefly, images captured from both the polar and mesocolpium (equatorial) viewpoints were delineated using ImageJ and subsequently subjected to elliptic Fourier transformations with the R package momocs (Bonhomme *et al*., 2014). The noise between the analyzed halves of images was removed with either ‘rm_sym’ or ‘rm_asym’ functions. The resulting 32 harmonic coefficients were then used in PCA, conducted independently for the polar and equatorial views. The first principal component (PC1) derived from each PCA was then employed as a descriptive parameter for pollen shape (Fig. S2). PC1 of the polar view accounted for 64.7% of the variance and effectively encapsulated the spectrum of mesocolpium shape at the poles. PC1 of the equatorial view explained 96.1% of the variance, showing a gradual transition from prolate‐spheroidal, through prolate and elliptic to perprolate, bone‐shaped morphologies.

### Molecular dataset

Sequences of nuclear rDNA ITS and plastid *matK*, *rbcL*, as well as introns in the *rpoC1*, *rps16*, and *rpl16* genes were either downloaded from NCBI using the MatPhylobi tool (https://github.com/hansiu/MatPhylobi) or generated specifically for this study. To generate the sequences, *c*. 20 mg of dried leaves from herbarium specimens was used for DNA isolation with the DNeasy Plant Mini Kit (Qiagen, Venlo, the Netherlands). The selected loci were amplified by PCR following previously established protocols and primers (Banasiak *et al*., 2016; Khederzadeh *et al*., 2017). Sanger sequencing was performed by Genomed S.A. (Warsaw, Poland), and the resulting reads were assembled using SeqMan Pro v.13.0.2 (DNAStar, Madison, WI, USA). All newly generated sequences have been deposited in GenBank (Supporting Information Table S1).

Our molecular dataset included 158 focal species of Apiales, along with an outgroup consisting of three representatives from other campanulid orders: *Helianthus annuus* L. (Asterales), *Lonicera chrysantha* Turcz. ex Ledeb. (Dipsacales), and *Quintinia verdonii* F.Muell. (Paracryphiales). After rooting, the outgroup sequences were removed from subsequent analyses. Each analyzed taxon was represented by at least two sequences, with marker coverage as follows: 89% for the *rps16* intron, 73% for the *rpl16* intron, 67% for nrDNA ITS, 62% for *rbcL*, 57% for *matK*, and 48% for the *rpoC1* intron.

### Phylogeny reconstruction

The sequences of individual markers were aligned using the G‐INS‐i algorithm, implemented in Mafft v.7.271 (Katoh & Standley, 2013). These alignments were then automatically trimmed with trimAl v.1.2rev59 (Capella‐Gutiérrez *et al*., 2009) with the –automated1 option. The resulting concatenated matrix, comprising a total of 5964 positions, was used for subsequent phylogenetic analyses. To determine the optimal partitioning scheme for the molecular dataset, the Bayesian Information Criterion (BIC) was employed with PartitionFinder v1.1.1 (Lanfear *et al*., 2012). The resulting scheme encompassed four partitions, each evolving according to distinct substitution models: (1) nrDNA ITS with SYM + I + G, (2) *matK*, *rpoC1* intron, and *rps16* intron with GTR + I + G, (3) *rbcL* with K80 + I + G, and (4) *rpl16* intron with GTR + G.

The phylogenetic tree was reconstructed using a parallel version of MrBayes v3.2.6 (Ronquist & Huelsenbeck, 2003) employing default priors and the inferred partitioning scheme. Two simultaneous analyses were conducted, each comprising 10 million generations, with one cold and three heated chains. The temperature for the heated chains was set to 0.3, and tree samples were collected every 100 generations. Tracer v.1.5 (Rambaut *et al*., 2018) was utilized to assess the convergence of the Markov Chain Monte Carlo chains.

### Climatic niche shifts

To construct the matrix of bioclimatic parameters, we first compiled information on the geographic distribution (latitude and longitude) for all occurrences of the focal species, based on data available at GBIF (gbif.org), with record categories: preserved specimen, observation, machine observation, and human observation. We then cleaned the dataset using the R package coordinatecleaner (Zizka *et al*., 2019) to remove problematic records, such as zero coordinates, sea coordinates, or coordinate–country mismatches. Finally, we extracted all bioclimatic variables (BIO1–BIO19) for each record from WorldClim (worldclim.org) at a spatial resolution of 2.5 arc‐min (*c*. 4 km^2^). The resulting matrix was standardized (z‐scaled), aggregated by taking the mean to obtain species‐level values, and used for PCA. Following scree‐plot analysis, we retained the first two principal components, accounting for a combined 65.4% of the total variation, as descriptors of climatic niche in Apiales (Table 1).

**Table 1:** Contribution of 19 bioclimatic variables to the first two principal components of climate space.

Bioclimatic variable	PC1 (23.4%)	PC2 (16.8%)
BIO1 = Annual Mean Temperature	**0.32**	−0.19
BIO2 = Mean Diurnal Range	0.02	−0.23
BIO3 = Isothermality	0.27	−0.12
BIO4 = Temperature Seasonality	**−0.30**	0.02
BIO5 = Max Temperature of Warmest Month	0.15	−0.28
BIO6 = Min Temperature of Coldest Month	**0.33**	−0.10
BIO7 = Temperature Annual Range	−0.28	−0.06
BIO8 = Mean Temperature of Wettest Quarter	0.17	−0.05
BIO9 = Mean Temperature of Driest Quarter	0.25	−0.20
BIO10 = Mean Temperature of Warmest Quarter	0.19	−0.23
BIO11 = Mean Temperature of Coldest Quarter	**0.34**	−0.14
BIO12 = Annual Precipitation	0.23	**0.32**
BIO13 = Precipitation of Wettest Month	0.27	0.16
BIO14 = Precipitation of Driest Month	0.09	**0.40**
BIO15 = Precipitation Seasonality	0.06	**−0.30**
BIO16 = Precipitation of Wettest Quarter	0.27	0.18
BIO17 = Precipitation of Driest Quarter	0.10	**0.40**
BIO18 = Precipitation of Warmest Quarter	0.17	0.28
BIO19 = Precipitation of Coldest Quarter	0.17	0.22

Boldface indicates traits with an important contribution, that is, loading exceeding 0.3.

To identify key evolutionary shifts in climatic niche occupation across Apiales, we applied multivariate OU processes implemented in the R packages l1ou (Khabbazian *et al*., 2016) and phylogeneticem (Bastide *et al*., 2018) to a 50% majority‐rule consensus tree from MrBayes. These approaches require no predefined grouping for shift detection, yet they vary in their strategies to achieve computationally tractable models. The *l1ou* method combines the OU process with LASSO (Least Absolute Shrinkage and Selector Operator; see Tibshirani, 1996) without retaining trait correlations in model fitting. In comparison, *PhylogeneticEM* employs a scalar OU model that accounts for trait correlation, albeit at the cost of the assumption that they share the same evolutionary rate toward respective optima. As both methods of shift detection require ultrametric trees (i.e. trees with equal distances from the root to the tips), we rescaled the consensus tree with nonparametric rate smoothing and penalized likelihood, as implemented in *TreePL* (Smith & O'Meara, 2012). The optimal smoothing parameter was chosen from a set of 10 values increasing tenfold from 0.00001 to 10 000. Parameter selection was based on cross‐validation analysis, with the age of the root node fixed at unity.

For the *l1ou* approach, we conducted the analysis using the phylogenetic Bayesian Information Criterion (pBIC) and assuming a fixed root. The pBIC is recognized for its conservative nature when estimating the number of shifts. To explore the effects of employing less stringent methods of shift detection, we performed additional reconstructions using the BIC. For each variant, we carried out 200 sequential iterations (all initiated from the same seed) of nonparametric bootstrap to evaluate the support for shifts at inferred nodes, based on their uncorrelated standardized residuals. For the *PhylogeneticEM* analysis, we used its default parameters.

### The effect of climate on pollen size evolution

To determine whether climate explains variation in pollen size across Apiales, we fitted phylogenetic generalized least squares (PGLS) models assuming evolution under Brownian motion (BM) or Ornstein–Uhlenbeck (OU) process using the R package phylolm with the formula: volume ~ PC1_clim_ + PC2_clim_. In the BM model, variation increases proportionally to the cumulative branch lengths from the root to the tips (Felsenstein, 1973, 1985) at a certain rate (σ), which serves as a signature of drift or stochastic alterations in selective pressure. Expanding upon BM, the OU model integrates the concept of stabilizing selection (Hansen, 1997; Butler & King, 2004), and features a long‐term mean‐reverting behavior (*α*), with traits oscillating around an optimal value (*θ*) over evolutionary time.

Although pollen grains are often approximated as ellipsoids for the purpose of volume estimation, we found this approach unsuitable due to the significant shape variation across Apiales. Instead, we extracted half of each pollen outline in the equatorial view, minimizing noise caused by slight asymmetry in the grains, and then revolved this half‐outline around the initial polar axis to produce a solid of revolution. The volume of the resulting solids was calculated using the disk method, employing the ‘trapz’ function from the pracma package (Borchers, 2019). Finally, these volumes were rescaled according to the known dimensions of the pollen grains based on their polar length.

### Multivariate analyses

In the next step, we tested whether distinct climatic regimes influenced evolutionary optima for wall thickness (WE/P and WP/P) and aperture morphology (ECT/P, ENDL/ENDW, and ENDL/P). The locations of climatic niche shifts identified by *l1ou*/*PhylogeneticEM* were used to define selective regimes, which were then mapped onto 100 randomly sampled trees from the posterior distribution of a MrBayes run.

For each tree, we fitted multivariate models of continuous trait evolution under several scenarios: a single‐rate BM model, a multi‐rate BM model with regime‐specific rates (BMM‐A for regimes inferred with *PhylogeneticEM*; BMM‐B for *l1ou*), and OU processes with either a single evolutionary optimum (OU1) or regime‐specific optima (OUM‐A and OUM‐B, analogous to BMM). To test for possible evolutionary lag between aperture morphology and wall thickness, we additionally fitted two OU models per regime framework, constraining the *α* matrix to represent scenarios in which (i) aperture morphology evolves toward optima defined by wall thickness (OUM‐A‐lag and OUM‐B‐lag) or (ii) wall thickness evolves toward optima defined by aperture morphology (OUM‐A‐lag2 and OUM‐B‐lag2). Specifically, the resulting matrix was symmetric within each trait set and triangular between sets, reflecting an interaction wherein one trait set influences the evolutionary trajectory of the other. All these analyses were performed using the ‘mvBM’ and ‘mvOU’ functions from the R package mvmorph (Clavel *et al*., 2015), employing both the standard log‐likelihood method (‘LL’) and RidgeArch penalization. The ancestral state at the root was assumed to correspond to the stationary distribution of the oldest regime (root = FALSE).

To investigate the presence of harmomegathy‐related syndromes in Apiales pollen architecture, we employed multivariate PGLS models using the same package. Specifically, we examined the predictive power of pollen wall thickness (WE/P and WP/P), nexine morphology (COSW/NP, COSL/P, NE/WE, and NP/WP), and aperture morphology (ECT/P, ENDL/P, and ENDL/ENDW) in explaining variation in the shape (polar and equatorial) of pollen grains. The model fitting was performed with the ‘mvgls’ function, assuming evolutionary processes under BM or OU models. We compared model fit using the Extended Information Criterion (EIC; Ishiguro *et al*., 1997; Kitagawa & Konishi, 2010) based on 1000 bootstrap replicates. To formally assess the significance of predictor variables under the best‐fitting model, we conducted type II phylogenetic MANOVA tests using Wilks's statistic and 9999 permutations.

## Results

### Phylogenetic framework

The Bayesian phylogeny of Apiales (Fig. S1) recovered relationships in agreement with the literature (Chandler & Plunkett, 2004; Andersson *et al*., 2006; Calviño & Downie, 2007; Nicolas & Plunkett, 2009; Downie *et al*., 2010; Wen *et al*., 2020; Xie *et al*., 2022). Pennantiaceae constitute a sister group to the remaining families classified within the order, followed by Torricelliaceae, Griseliniaceae, and suborder Apiineae, uniting Myodocarpaceae, Pittosporaceae, Araliaceae, and Apiaceae. Within Apiaceae, a clear delineation into four well‐established subfamilies – Mackinlayoideae, Azorelloideae, Saniculoideae, and Apioideae – can be observed, along with three genera *incertae sedis*: *Platysace* Bunge (recovered as a sister to Mackinlayoideae), *Hermas* L. (an isolated lineage arising before the divergence of Azorelloideae), and *Klotzschia* Cham. (sister to Saniculoideae + Apioideae). The phylogenetic patterns in Apioideae also align with their current intrafamilial classification.

The backbone and nearly all deep branches of the phylogenetic tree were reconstructed with high posterior probability (PP > 0.95). Uncertainties exist regarding the putative sister group of Apiaceae (either Araliaceae or Pittosporaceae), the affinity of the ‘North American *Ligusticum* clade’, *Platysace*, *Hermas*, and *Klotzschia*, as well as the branching order of some clades within Apioideae (Bupleureae, ‘*Pleurospermopsis* clade', Apieae, Pimpinelleae, Coriandereae, and Selineae+Echinophoreae).

### Shifts in climatic niche

The PCA of bioclimatic variables explained 40.2% of the cumulative variance in climatic niche occupation across Apiales (Table 1). The first principal component (PC1), which accounts for 23.4% of total variation, is strongly correlated (with absolute values of loadings equal to or exceeding 0.3) with variables describing temperature seasonality and, particularly, the severity of winter months. This includes annual mean temperature (BIO1; 0.32), temperature seasonality (BIO4; −0.30), mean temperature of the coldest month (BIO6; 0.33), and mean temperature of the coldest quarter (BIO11; 0.34). The second principal component (PC2), which accounts for 16.8% of the total variation, is positively correlated with annual precipitation (BIO12; 0.32), precipitation of the driest month (BIO14; 0.40), and precipitation of the driest quarter (BIO17; 0.40), and negatively correlated with precipitation seasonality (BIO15; −0.30).

**Fig. 2:**
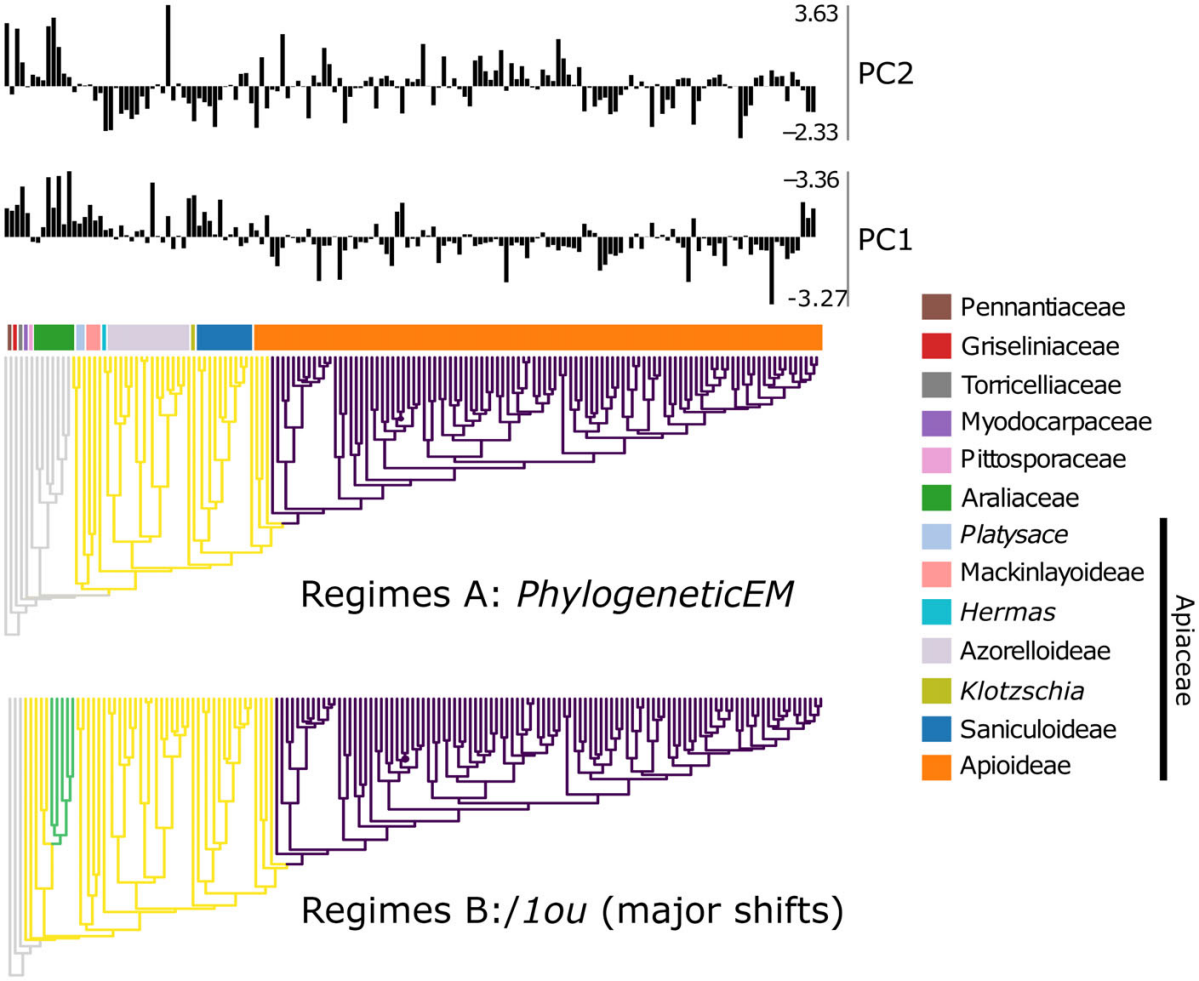
Major climatic niche shifts according to *PhylogeneticEM* and *l1ou* based on BIC and pBIC criteria. Color changes on the 50% majority‐rule consensus tree indicate the occurrence of shifts. The bar plots represent species‐wise values of PC1 and PC2, reconstructed from 19 bioclimatic variables obtained from WorldClim.

Nonumbelliferous Apiales occupy a climatic niche marked by relatively high values of both PC1 (warmer climates with lower temperature seasonality) and PC2 (wetter climates with less seasonal precipitation), consistent with their predominantly tropical and subtropical distribution. Earlier‐diverging Apiaceae (Mackinlayoideae, Azorelloideae, Saniculoideae, and African lineages of Apioideae), while still favoring regions with less seasonal temperature variation, tend to grow in distinctly drier habitats compared to the previous group. Reflecting their distribution in the Northern Hemisphere, core apioids (subfamily Apioideae following the divergence of the tribe Bupleureae) are found in habitats with varied precipitation patterns and high temperature seasonality (Fig. 2).

*PhylogeneticEM* proposed only two shifts: one at the base of Apiaceae and another within the core Apioideae (following the divergence of the tribe Chamaesieae). In the *l1ou* analysis, both detection criteria (BIC and pBIC) identified three major shifts: (1) at the base of the suborder Apiineae (including Pittosporaceae, Myodocarpaceae, Araliaceae, and Apiaceae), (2) within the *‘Eleutherococcus*‐*Dendropanax*‐*Schefflera* clade’ (Araliaceae), and (3) within the core apioids. Additionally, two smaller shifts were reconstructed in the genus *Arracacia* Bancr. and within the tribe Oenantheae, along with singleton shifts at the branches leading to *Azorella haastii* (Hook.f.) Drude, *Bupleurum aureum* Fisch. ex Hoffm., and *Pachypleurum alpinum* Ledeb. All these shifts were supported by bootstrap analysis (> 70).

We focused on two different scenarios of climatic niche evolution for downstream analyses (Figs 2, S3). Hypothesis A, informed by the results of *PhylogeneticEM*, posited two regime shifts: in early‐diverging Apiaceae, and in core apioids. Hypothesis B tested the presence of major niche shifts deduced from *l1ou*, with three shifts: in the suborder Apiineae, in the ‘*Eleutherococcus‐Dendropanax‐Schefflera* clade’, and in core apioids. After initial testing, we excluded the smaller/singleton *l1ou* shifts due to the computational burden and issues with Hessian optimization convergence.

### Impact of climate on pollen size

**Fig. 3:**
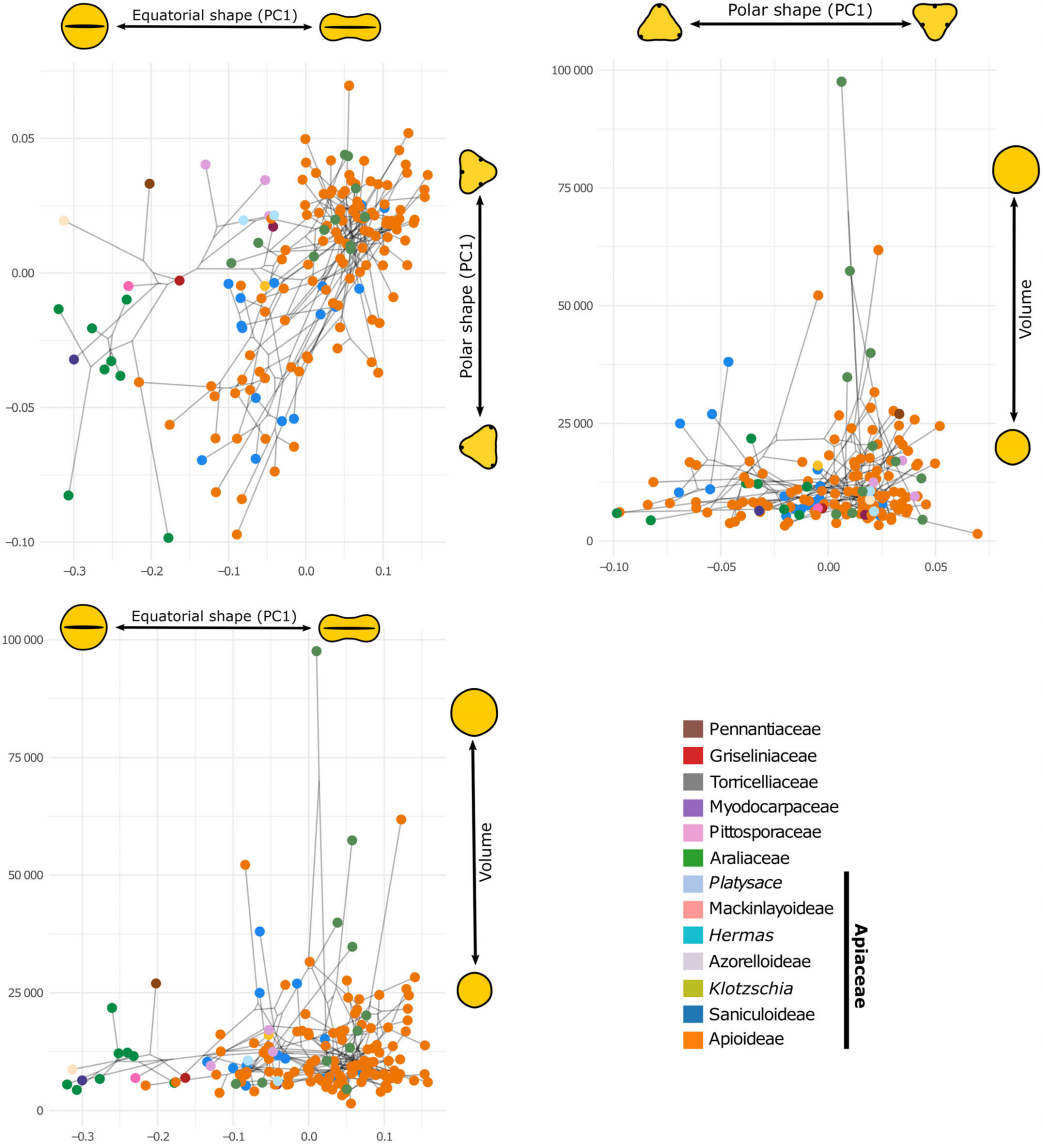
Phylomorphospace of pollen shape and size in Apiales. The plots show the position of species based on polar and equatorial shape (top‐left), polar shape and volume (top‐right), and equatorial shape and volume (bottom).

Climate showed no significant effect on pollen size in Apiales (Fig. S4). Models assuming evolution under the OU (stabilizing selection) process were favored over BM (drift or stochastic variation in selection) across all 100 trees; however, none of them revealed a significant association between PC1, PC2, and the volume of pollen grains. The reconstructed phylomorphospace shows considerable variation in pollen size across both early‐diverging, tropical/subtropical families of Apiales and the predominantly temperate Apiaceae, with no clear association between volume and phylogeny or overall grain shape (Fig. 3). Notably, the greatest size diversity is found within the predominantly herbaceous core apioids, which thrive in open habitats of the Northern Hemisphere. This diverse group includes both the smallest (*Ammoides pusilla*, *c*. 1500 μm^3^) and the largest (*Turgenia latifolia*, over 90 000 μm^3^) pollen grains analyzed in the study.

### Evolutionary optima in pollen wall and aperture

**Fig. 4:**
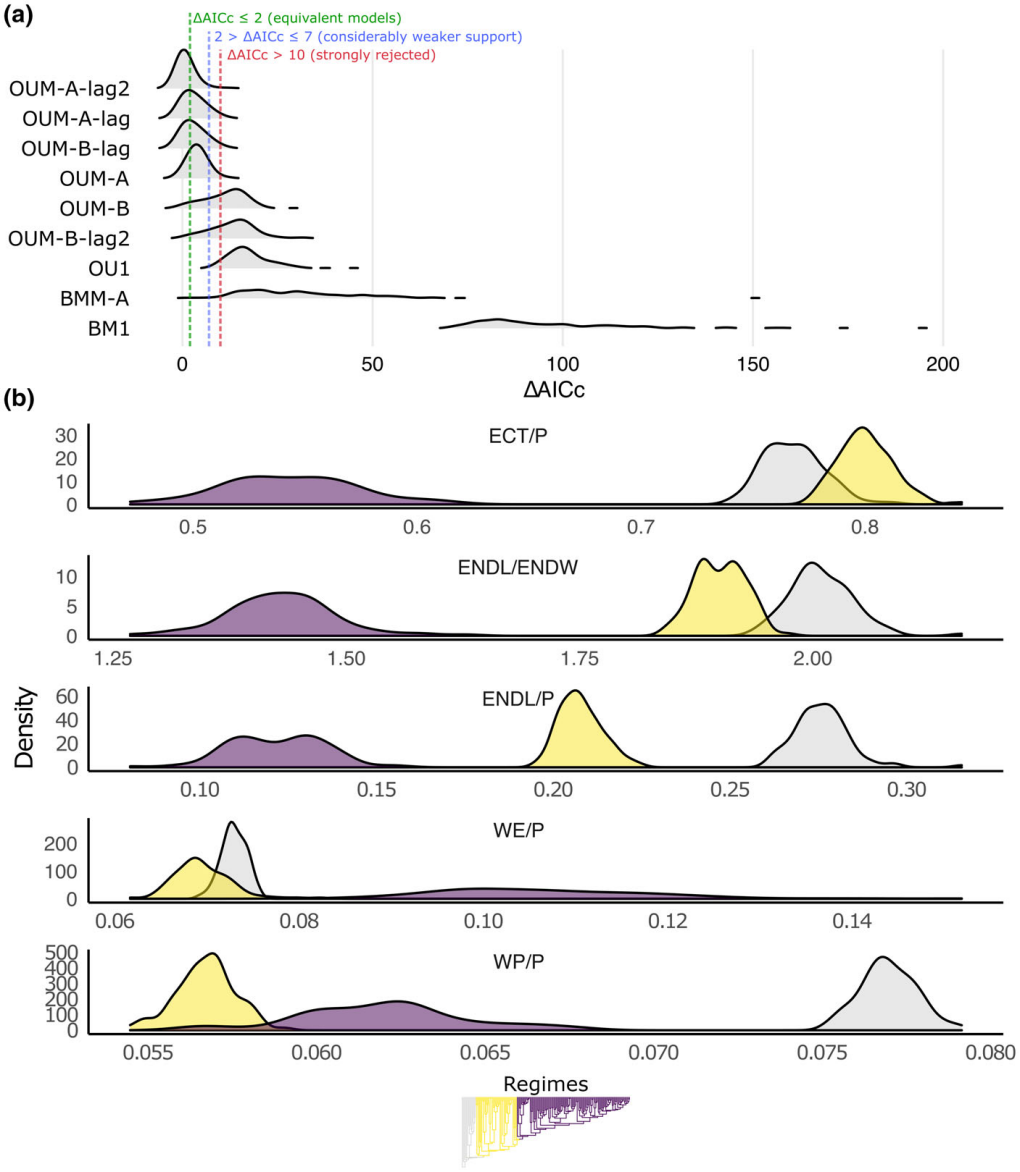
Results of multivariate model fitting for aperture and pollen wall thickness across 100 posterior trees from MrBayes. (a) Model support expressed as ΔAICc distributions for all fitted models, shown as ridgeline density plots. Lower ΔAICc values indicate better model performance, and the width and shape of each ridge reflect how consistently a given model is supported across the posterior trees. (b) Probability density estimates for evolutionary optima based on the OUM‐A‐lag2 model. ECT/P, ectoaperture length relative to polar length; ENDL/P, endoaperture length relative to polar length; ENDL/ENDW, endoaperture elongation; WE/P, wall thickness at the equator relative to polar length; WP/P, wall thickness at poles relative to polar length. The phylogenetic tree at the bottom represents the selective regimes (i.e. shifts in climatic niche; see Fig. 2).

The multivariate model fitting for aperture and pollen wall morphology (Fig. 4a) showed a strong preference (92 out of 100 trees) for multi‐rate OU models with three distinct selective regimes, corresponding to climatic niche shifts reconstructed by PhylogeneticEM. Additionally, our analyses provide strong support for an evolutionary lag, with pollen wall thickness responding first, followed by aperture morphology. The model that assumed a delayed response in aperture morphology compared to pollen wall thickness (OUM‐A‐lag2) was the best fit for 42 trees. For 49 trees, however, the analysis remained inconclusive regarding the direction of the evolutionary lag, with ΔAICc < 2 between the two best‐fitting models: OUM‐A‐lag1 or OUM‐A‐lag2. BMM models with regimes corresponding to major l1ou shifts (BMM‐B) failed to reach a stable solution, despite multiple attempts using different likelihood computation methods.

Our results support the view that shifts in climatic niche are major drivers of aperture and pollen wall evolution in Apiales and suggest (though not unanimously) that the former lags behind the latter. Moreover, phylogenetic half‐lives (i.e. the time required for a trait to evolve halfway toward its optimal value in response to selective pressures) estimated under OUM‐A‐lag2 indicate that while all analyzed traits rapidly approach their respective optima, the evolution of aperture morphology (ECT/P = 0.09 ± 0.02; ENDL/P = 0.15 ± 0.02; ENDL/ENDW = 0.17 ± 0.03) is noticeably slower than that of pollen wall thickness (WE/P = 0.07 ± 0.01; WP/P = 0.05 ± 0.01).

The estimated evolutionary optima (Fig. 4b) suggest that the radiation of Apiaceae from ancestral subtropical/tropical habitats was accompanied by a reduction in aperture size. Although the optima for ectoaperture length and endoaperture elongation in early‐diverging Apiaceae partially overlap with those of early‐diverging Apiales, a trend toward shorter endoapertures is already evident. The diversification of core apioids in the Northern Hemisphere corresponds to more pronounced shifts, favoring short colpi (< 0.6 of polar length) and endoapertures. In comparison with apertures, evolutionary patterns in pollen wall thickness are more complex. Pollen grains in early‐diverging Apiales possess a relatively thick and uniform sporoderm. The divergence of Apiaceae is associated with strong selection for a thinner wall at the poles, likely reflecting an overall change in pollen shape. Subsequently, in core apioids, the pollen wall thickened, particularly at the mesocolpium, though polar wall thickness remains lower than in early‐diverging Apiales.

### Morphological correlations in pollen architecture

**Fig. 5:**
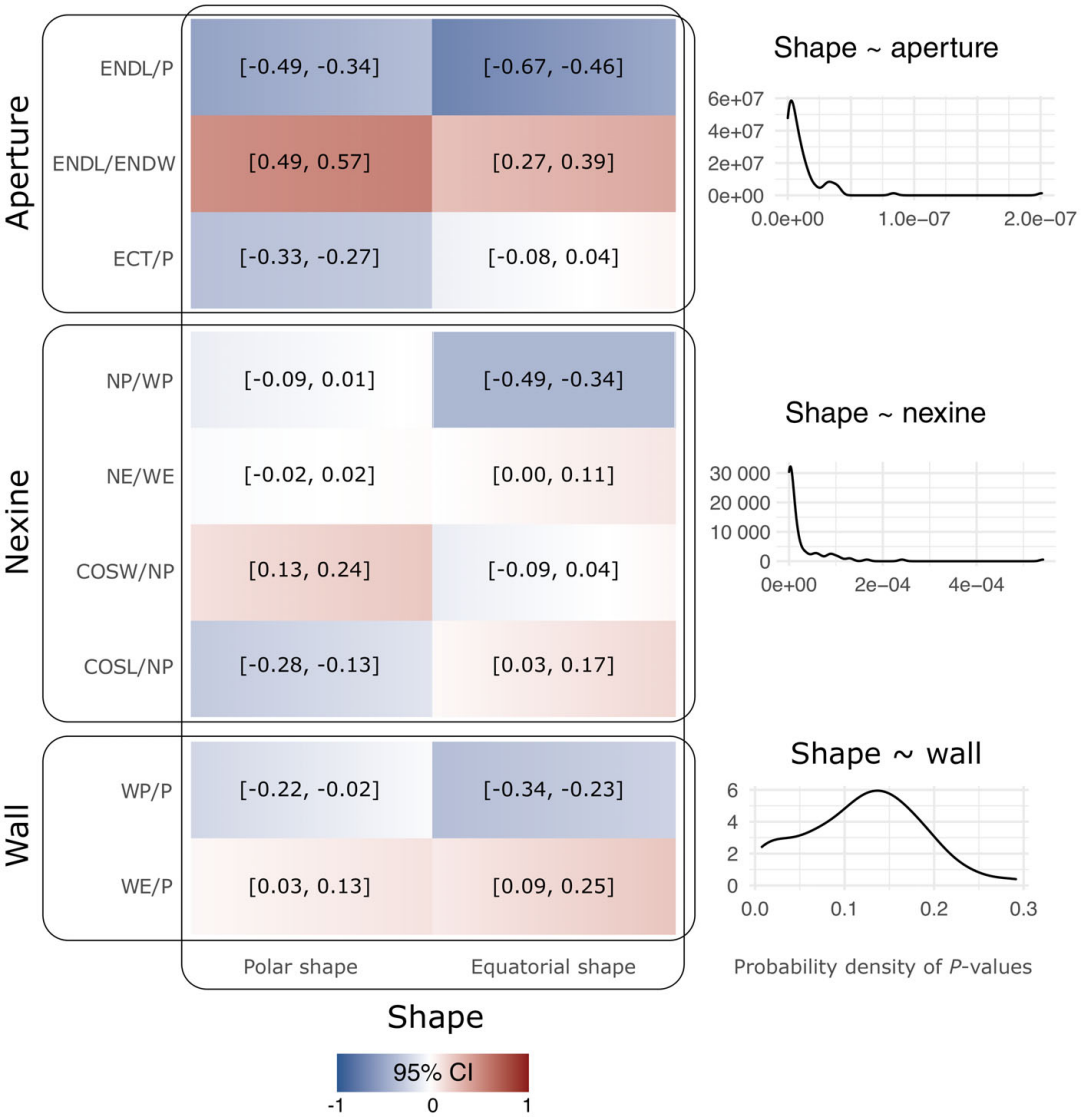
Results of multivariate PGLS model fitting across 100 posterior trees from MrBayes. The heatmap shows the 95% confidence intervals for the correlation coefficient estimates, while the line plots on the right depict the probability density distributions of p‐values obtained from phylogenetic MANOVA tests, evaluating associations between shape and aperture morphology (upper panel), nexine stratification (middle panel), and wall thickness (lower panel).

Our analyses identified the OU model as a better fit compared to the BM model across all 100 analyzed trees. The phylogenetic MANOVA detected a significant correlation between pollen shape and both aperture morphology and nexine stratification (Fig. 5). However, the association between pollen shape and wall thickness remained largely inconclusive, likely influenced by minor topological differences among the analyzed trees, as significance was recovered only in some cases. Equatorially elongated grains with sharply bent mesocolpia in polar view, indicating a dynamic function and considered an apomorphic morphology, show a strong correlation with shorter (ENDL/P) but more elongated endoapertures (ENDL/ENDW) and thinner nexine at the poles (NP/WP). A moderate negative correlation was also detected between the polar shape of pollen grains and shorter ectoapertures (ECT/P). The remaining correlations detected in the study, although significant, are weak or negligible.

## Discussion

The structure of the sporoderm reflects a delicate equilibrium between its dynamic function in accommodating volume changes in harmomegathy and its protective functions. Our study in Apiales reveals that macroevolutionary trends in pollen morphology are consistent with established theoretical models (Katifori *et al*., 2010; Božič & Šiber, 2020) and with observation‐based interpretations (Payne, 1972, 1981; Halbritter & Hesse, 2004; Volkova *et al*., 2013; Banks & Rudall, 2016). Importantly, the inferred functional coupling between apertures, wall thickness, and shape aligns with the notion that aperture architecture is a major determinant of harmomegathic responses and therefore one of the key drivers of pollen diversity across angiosperms (Pacini & Franchi, 2020). Our morphometric analyses and correlations with bioclimatic variables suggest that in Apiales, the shift from tropical to temperate biomes led to a gradual selection for thicker sexine, followed by the reduction of compound apertures. These alterations, in turn, drove subsequent changes in pollen shape in core apioids to enhance harmomegathal efficiency.

### Adaptation to changing climate affects pollen architecture, but not its size

Our study revealed that the size of pollen in Apiales does not correlate with temperature or precipitation gradients across latitudes. This finding aligns with the work of Kriebel *et al*. (2017) and Wei *et al*. (2023), who also found no association between climate and pollen size. Although these results may seem surprising, given the assumption that larger, more spherical grains reduce water evaporation by reducing the surface‐to‐volume ratio, Ejsmond *et al*. (2015) reported a positive correlation between pollen size and temperature, regardless of potential evapotranspiration. They further suggested that this reflects the relationship between higher temperatures and increased pollinator activity. We therefore hypothesize that the lack of correlation between pollen size and temperature in Apiales can be attributed to pollination biology as well. Owing to their open, umbel‐like inflorescences, abundant flowering, and unrestricted access to floral rewards, Apiales – particularly umbellifers – are characterized by highly generalized pollination systems. When combined with widespread andromonoecy and a high investment in male reproductive function (Spalik, 1991), these traits suggest that competition for stigmas is especially intense in this group, constraining the evolution of pollen size, particularly at the lower end. Accordingly, most pollen grains in Apiales are medium to large in size, typically ranging from 30 to 50 μm in polar length.

A second, nonexclusive explanation for the lack of a macroevolutionary correlation between pollen size and desiccation intensity is the predominantly trinucleate condition of Apiales pollen. Compared to binucleate pollen, trinucleate grains are more susceptible to drying due to their thinner exine, lower carbohydrate reserves, and higher metabolic activity (Hoekstra & Bruinsma, 1975; Williams & Brown, 2018). These factors collectively shorten pollen lifespan and may favor strategies that avoid desiccation risk rather than mitigate it physiologically, such as restricting pollen release to periods of lower evaporative stress (Leduc *et al*., 1990; Muccifora *et al*., 2003).

While pollen size shows no climatic signal, our results demonstrate that climatic niche strongly influences pollen architecture. Indeed, the results of multivariate, multi‐regime model fitting indicate that climatic niche affects optima for aperture morphology and pollen wall thickness in Apiales. Specifically, thicker walls and smaller apertures were consistently favored in drier, more seasonal environments, likely reflecting adaptation to reduced moisture availability by limiting water loss during transport. Variation in aperture and wall traits can be attributed to an evolutionary trade‐off between germination rate and protection against desiccation and UV radiation (Edlund *et al*., 2004; Franchi *et al*., 2011; Pacini & Hesse, 2012). For instance, submerged aquatic plants with underwater pollination, such as Zosteraceae or Cymodoceaceae, produce recalcitrant exineless pollen (Ackerman, 1995), and similar reductions in exine have evolved in aquatic *Callitriche* L. species (Cooper *et al*., 2000).

Comparable trends occur across other plant lineages. For example, Nogué *et al*. (2022) observed an increased prevalence of grains with thick walls and colpi (vs pores limiting harmomegathal movements) in drier regions of the Canarian laurel forest. Similarly, Fatmi *et al*. (2020) linked aperture morphology to aridity in *Atriplex halimus* L. Notably, the reduction in aperture size and number has also been observed as a common trend in xerophytic plants (Thanikaimoni, 1986). In Apiaceae, similar adaptations have independently evolved in the thistle‐like members of the tribe Echinophoreae; their thick‐walled, uniquely dicolporate pollen likely serves as protection against increased desiccation intensity in dry habitats, such as coastal dunes. Our findings mirror these comparative patterns, demonstrating that reduced aperture size and increased wall thickness are strongly associated with environments experiencing greater desiccation stress, consistent with convergent adaptive responses across distantly related lineages.

### Evolution of pollen shape reflects functional constraints

Since our study relied on acetolysed material obtained from herbarium specimens, most of which were decades old, direct observation of harmomegathal patterns and their association with morphological data was not feasible. While there are instances where it is possible to infer modes of infolding based on the potential distribution of weak points in the sporoderm, Halbritter & Hesse (2004) demonstrated that pollen that appears superficially similar when hydrated can exhibit notable differences after drying. To address these limitations, we supplemented our dataset by examining photographs of both hydrated and dry grains from PalDat (paldat.org) and consulting the primary literature, which often includes scanning electron microscope (SEM) images of pollen following acetolysis. Through this combined approach, we identified several distinct harmomegathal syndromes in Apiales that align closely with theoretical predictions based on soft shell modeling (Katifori *et al*., 2010; Božič & Šiber, 2020).

Axially elongated apertures behave as preferred sites for predictable infolding during dehydration, with columellae serving as structural reinforcements counteracting increased tension (Blackmore & Barnes, 1986; Crane, 1986). However, as the colpi shorten, their mesocolpium‐mediated closing effectiveness becomes gradually compromised (Katifori *et al*., 2010). Božič & Šiber (2020, fig. 6) demonstrated that early indicators of this transition include the emergence of a distinctly lobate shape in dry pollen (see also Halbritter & Hesse, 2004), followed by progressive exine flattening as the aperture loses its harmomegathal function.

The importance of aperture morphology for stabilizing shape‐change patterns likely underlies the observed lag between modifications of pollen wall structure and changes in colpi/ora morphology in Apiales. We propose that an overall thinning of the sporoderm – particularly in the polar region – at the base of Apiaceae increased the elasticity of the pollen wall and enhanced its ability to accommodate volumetric changes during hydration without rupturing. Only once this greater mechanical flexibility had evolved could substantial aperture modifications, such as the progressive shortening of colpi, occur without compromising pollen integrity. In core apioids, subsequent uneven thickening of the sexine in the interapertural regions may then have further stabilized harmomegathal patterns by defining the distribution of weak and rigid zones within the wall.

Knowledge of pollen morphology in early‐diverging Apiales remains limited due to a lack of comparative studies, especially in the three families at the base of the order. Before the APG IV (Chase *et al*., 2016), representatives of Torricelliaceae and Griseliniaceae were often placed within a broadly defined Cornales or treated as monogeneric orders within Cornanae, while *Pennantia* J.R.Forst. & G.Forst. was classified within Icacinaceae (Takhtadzhian, 1997). The palynological studies of *Pennantia* and *Griselinia* Scop. (Dahl, 1952; Chao, 1954; Tseng, 1980) suggest that their infolding patterns may be similar to those at the base of Apiineae; however, we were unable to find any photographs or drawings of dry pollen to confirm or refute this hypothesis. Torricelliaceae, on the other hand, are morphologically distinct within Apiales, with thick‐walled, nearly spherical pollen with highly reduced apertures (Ferguson & Hideux, 1978; Tseng, 1980), making direct comparisons even more difficult.

**Fig. 6:**
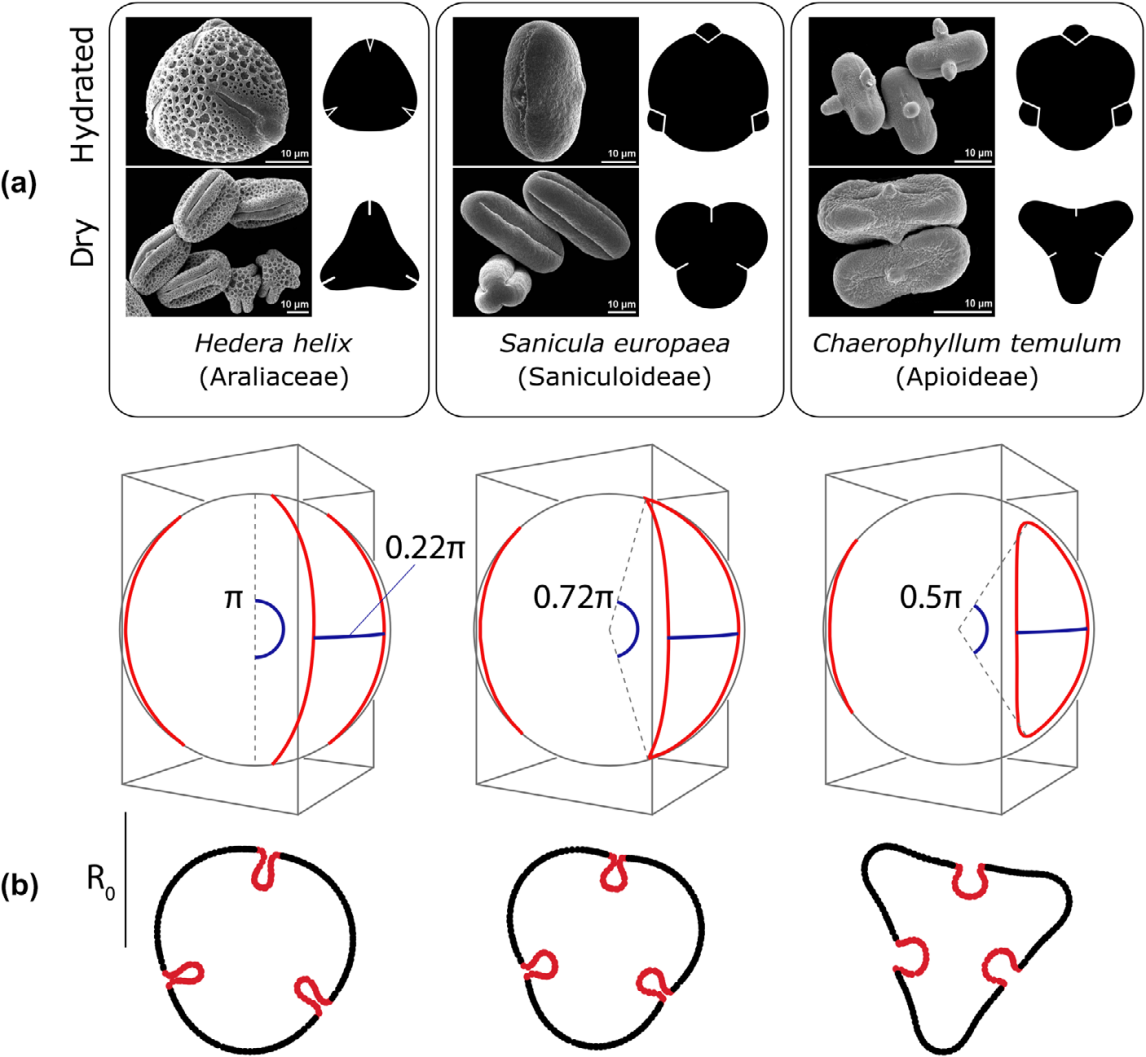
Harmomegathal patterns of pollen in Apiales (a) contrasted with theoretical predictions (b) by Božič & Šiber (2020). Most early‐diverging Apiales produce short, thin‐walled grains with extended ectoapertures and pronounced endoapertures (left, *Hedera helix*). Their harmomegathal movements are guided by buckling of the apertures and mesocolpia, resulting in a polar outline with apertures situated at angles. Prolate elliptic pollen with slightly reduced apertures (70–80% of the polar distance), as observed in Saniculoideae, some Azorelloideae, and certain apioids, exhibits a lobed pattern in dry grains (middle, *Sanicula europaea*), primarily driven by infolding along the colpi. Core apioids, such as *Chaerophyllum temulum*, display pollen with very short apertures and unevenly thickened exine, producing a triangular polar view with lateral apertures when dry (right). SEM images sourced from paldat.org.

In the three early‐diverging families of suborder Apiineae (Araliaceae, Pittosporaceae, and Myodocarpaceae) pollen is usually columellate, with perforate, reticulate, or striato‐reticulate ornamentation (Tseng & Shoup, 1978; Philipson & Stone, 1980; Tseng *et al*., 1983; Wen & Nowicke, 1999; Fiaschi *et al*., 2010). This diverse exine patterning likely helps maintain a stable pattern of volumetric changes (Katifori *et al*., 2010; Volkova *et al*., 2013; Banks & Rudall, 2016; Matamoro‐Vidal *et al*., 2016) which, in Araliaceae, occurs mainly via buckling and de‐buckling of the mesocolpium (Fig. 6, left). Overall similar pollen morphology and harmomegathal patterns are also found in Mackinlayoideae and early diverging Apioideae, but with more prominent equatorial elongation and uniform striate or regulate ornamentation, as is typical for Apiaceae. In Saniculoideae and likely most Azorelloideae (Cerceau‐Larrival, 1974; photographs taken from acetolysed grains), sealing of pollen happens predominantly through infolding of colpi (Fig. 5, center), producing a characteristic three‐lobed pattern in dry grains (Halbritter & Hesse, 2004; Božič & Šiber, 2020). This mechanism results from a uniform and relatively thin columellate exine.

While colpus‐mediated infolding also occurs in Apioideae, most representatives of this diverse subfamily have thickened, columellate exine along the equator (at mesocolpia) and shortened apertures that cannot seal as easily as in species with long, slit‐like colpi. In those species, at the time of dehydration, entire apertural areas come closer to each other in a process resembling ‘sinking’ inwards of the grain (Fig. 5, right). Although based on a different architectural solution, a similar mechanism has been described for lophate pollen of Asteraceae (Blackmore, 1982; Siljak‐Yakovlev *et al*., 1986) in which lacunae (depressed areas of sporoderm surrounded by ridges) facilitate volume changes in the presence of short and largely immobile colpi. Both of these harmomegathal pathways are frequently associated with intine bulging (most extreme in *Chaerophyllum temulum* L., Fig. 5), which can be considered as an additional mechanism to cope with swelling during pollen rehydration (Božič & Šiber, 2022).

Our study highlights the potential of combining morphometric analyses with advanced statistical tools and expanding biodiversity databases to understand how different selective regimes shape morphological diversity within and across plant lineages. Although our research focuses on a limited subset of extant Apiales diversity, it provides a valuable framework for future analyses that incorporate fossil taxa. Leveraging the extensive palynomorph fossil record alongside deep learning approaches (Adaïmé *et al*., 2024), the fossilized birth–death model (Heath *et al*., 2014), and other total‐evidence approaches (Ronquist *et al*., 2012; Zhang *et al*., 2016; Gavryushkina *et al*., 2017) could address key questions about the early diversification of flowering plants (Sauquet & Magallón, 2018; Sauquet *et al*., 2022). These methods have already provided significant insights into the evolution of fruits (Larson‐Johnson, 2016) and flowers (López‐Martínez *et al*., 2023), and are now being applied to pollen (Bacon *et al*., 2022; Woutersen *et al*., 2023).

## Competing interests

None declared.

## Author contributions

JB and ŁB designed the study and compiled the data. JB analyzed the data and led the writing of the paper. ŁB, JMB and KS revised multiple drafts of the manuscript.

## Disclaimer

The New Phytologist Foundation remains neutral with regard to jurisdictional claims in maps and in any institutional affiliations.

## Supporting information


**Fig. S1** Phylogenetic tree for species used in this study.
**Fig. S2** PCA of pollen shape harmonic coefficients.
**Fig. S3** Climatic space for selective regimes.
**Fig. S4** Results of evolutionary model fitting for association between climate and pollen size.
**Table S1** Sequences used in this study.
Please note: Wiley is not responsible for the content or functionality of any Supporting Information supplied by the authors. Any queries (other than missing material) should be directed to the *New Phytologist* Central Office.

## Data Availability

All data files, including molecular datasets, phylogenetic trees and code used in the analysis are available for download at the corresponding author's GitHub page (https://github.com/JaJeBacz/Apiales_pollen).
