# Supplementary material for: Climate adaptation and functional constraints drive pollen evolution in Apiales
Source: New Phytol. 2025 Dec 15;249(5):2574–87. doi: 10.1111/nph.70824 (PMC12873509; doi:10.1111/nph.70824)
Supplement: Supplementary file 1 — Fig. S1 Phylogenetic tree for species used in this study. Fig. S2 PCA of pollen shape harmonic coefficients. Fig. S3 Climatic space for selective regimes. Fig. S4 Results of evolutionary model fitting for association between climate and pollen size. Table S1 Sequences used in this study. Please note: Wiley is not responsible for the content or functionality of any Supporting Information supplied by the authors. Any queries (other than missing material) should be directed to the New Phytologist Central Office. [file NPH-249-2574-s001.docx]

## *New Phytologist* Supporting Information

Article title: **Climate adaptation and functional constraints drive pollen evolution in Apiales**

Authors: Jakub Baczyński, Krzysztof Spalik, John M. Burke, Łukasz Banasiak

Article acceptance date: 21 November 2025

The following Supporting Information is available for this article:

**Fig. S1** Phylogenetic tree for species used in this study.

**Fig. S2** PCA of pollen shape harmonic coefficients.

**Fig. S3** Climatic space for selective regimes.

**Fig. S4** Results of evolutionary model fitting for association between climate and pollen size.

**Table S1** Sequences used in this study.

**Fig. S1** Maximum clade credibility tree for 161 species (158 Apiales and three outgroup taxa), reconstructed using Bayesian approach. Branch values indicate posterior probability and only branches with low support (< 0.95) were plotted.

**
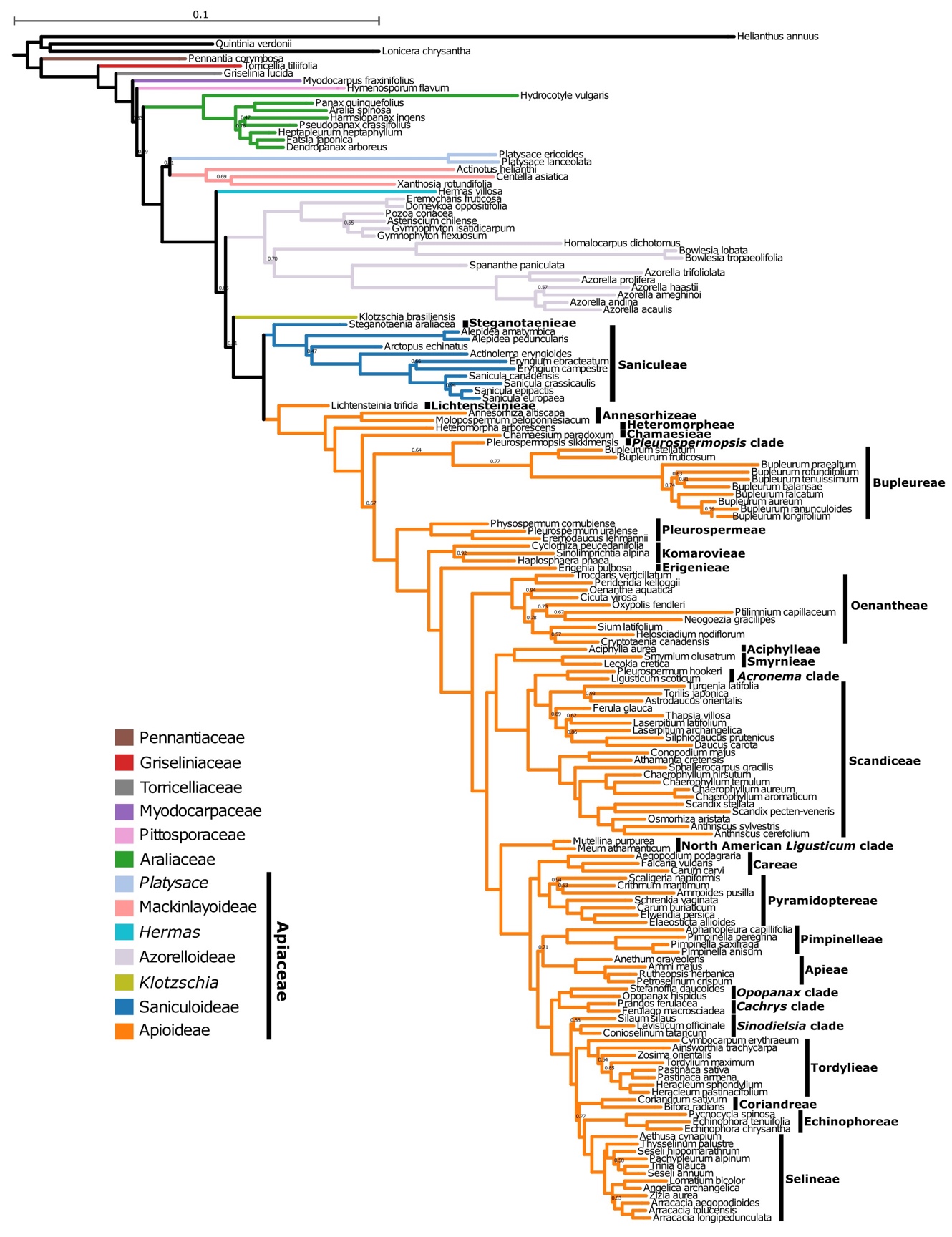
**

**Fig. S2** PCA of pollen shape based on 32 harmonic coefficients, reconstructed for equatorial and polar view using R package *Momocs*. The first principal components from both views were used as continuous shape descriptors in downstream analyses.

**
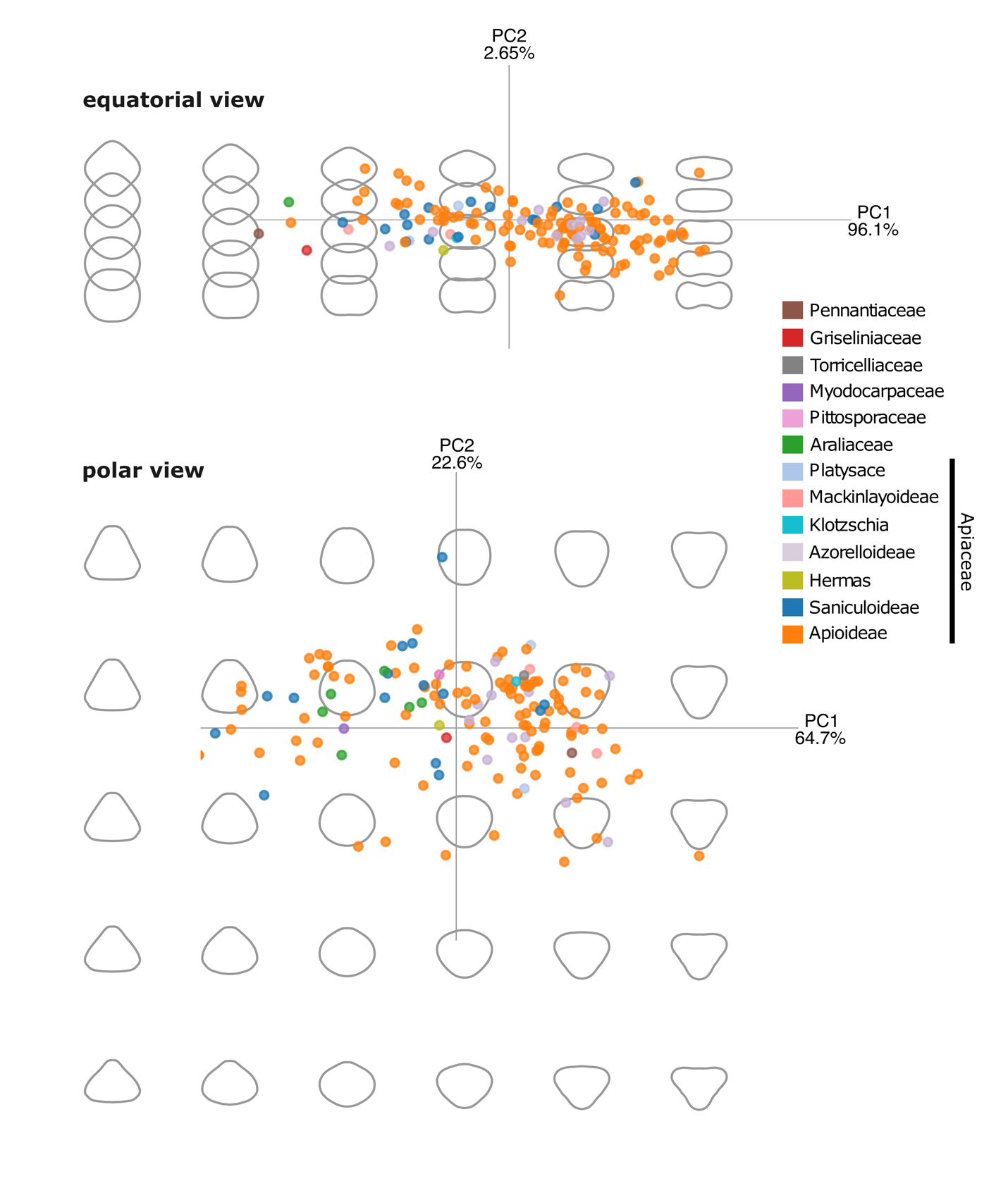
**

**Fig. S3** Climatic space associated with selective regimes inferred using *l1ou* (upper left) and *PhylogeneticEM* (upper right). The bottom panel shows eigenvectors of 19 bioclimatic variables based on their loadings on the first two principal component axes; variables with major contributions (> 0.3) are indicated in bold.

**
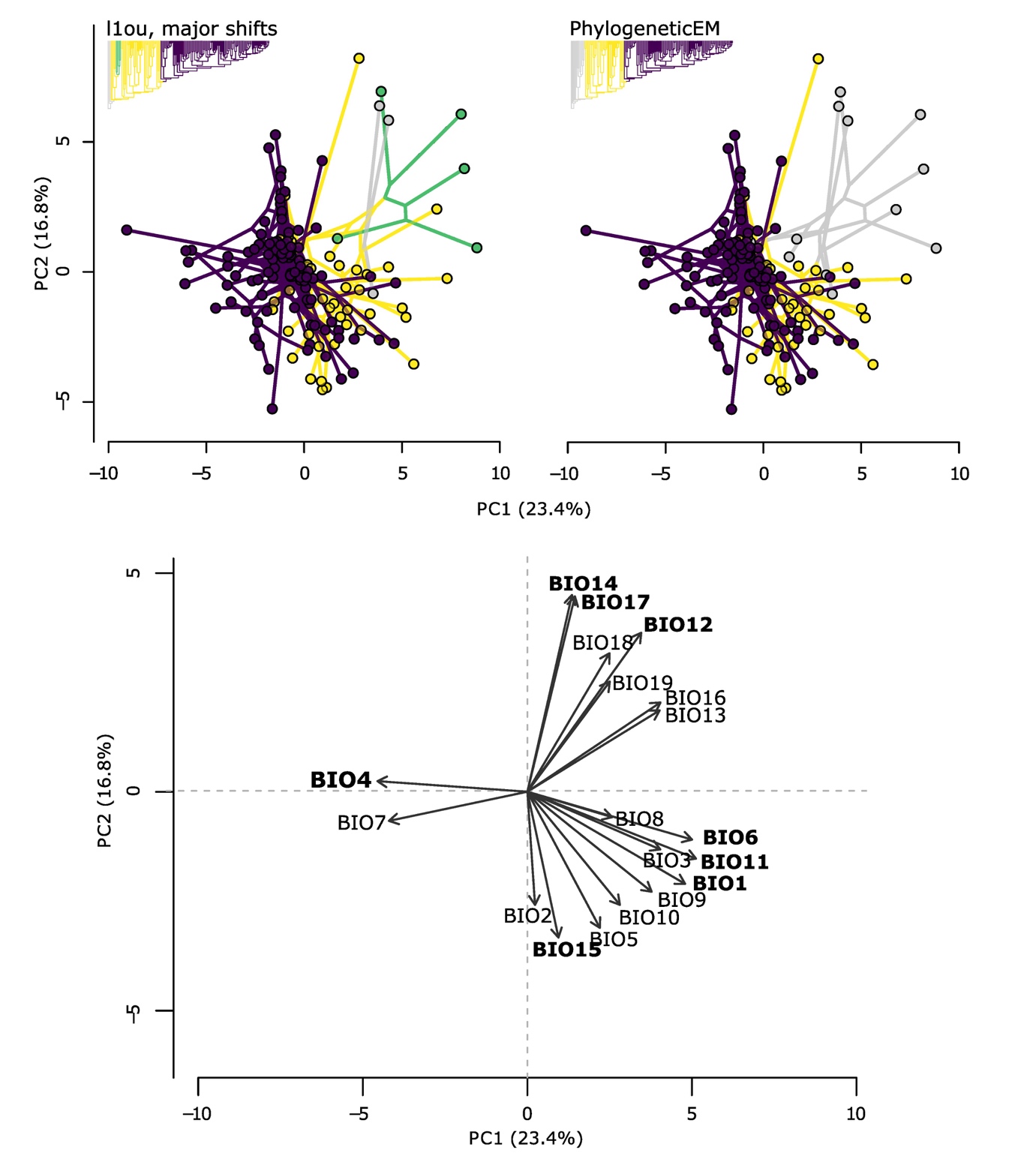
**

**Fig. S4** Results of Brownian Motion (BM) and Ornstein–Uhlenbeck (OU) model fitting for the association between climate and pollen size, summarized across 100 Bayesian posterior trees. The bottom panels show probability density distributions of p-values from Wald tests evaluating the relationship between pollen size and either PC1 or PC2 of the bioclimatic variables.

**
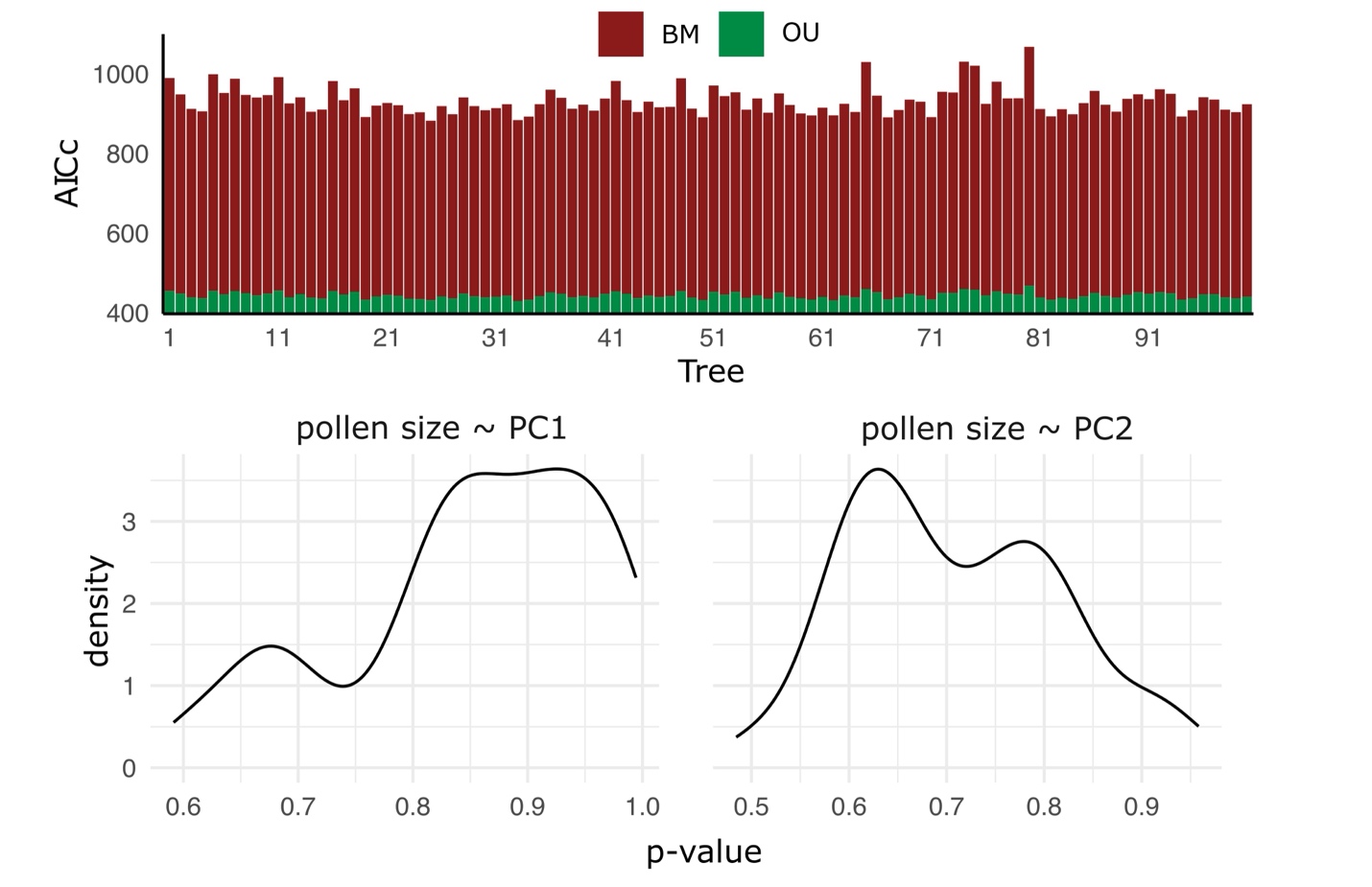
**

**Table S1** Accession numbers correspond to GenBank identifiers. For nuclear internal transcribed spacer (ITS) sequences, two identifiers in parentheses indicate that ITS1 and ITS2 regions were obtained separately. Accession numbers in bold indicate sequences newly generated in this study.

| Species | rps16 | rpoC1 | matK | rbcL | rpl16 | ITS |
| --- | --- | --- | --- | --- | --- | --- |
| *Aciphylla aurea* | **ON107378** |  | U58541.1 | AY188411.1 | GQ243901.1 | U72377.1 |
| *Actinolema eryngioides* | DQ832336.1 |  |  |  | GQ243948.1 |  |
| *Actinotus helianthi* | DQ133826.1 |  | AF271760.1 | DQ133797.1 | GQ243879.1 |  |
| *Aegopodium podagraria* | DQ133827.1 |  | JN895997.1 | KM360614.1 | GQ243904.1 | MT937105.1 |
| *Aethusa cynapium* | AF110539.1 | U36278.1 | KJ204433.1 | KM360617.1 | AF094406.1 | GQ862376.1 |
| *Ainsworthia trachycarpa* |  |  | KX401336.1 |  |  | EU169243.1 |
| *Alepidea amatymbica* | DQ832338.1 | **ON107361** |  |  |  |  |
| *Alepidea peduncularis* | EU168945.1 |  | ON981939.1 |  | GQ243950.1 |  |
| *Ammi majus* | AF164814.1 | MT513169.1 | HM850716.1 | JQ230996.1 | MW036669.1 | JQ230973.1 |
| *Ammoides pusilla* | **ON107463** | **ON107348** | **ON086530** |  |  | HE602454.1 |
| *Anethum graveolens* | AF110542.1 | U36281.1 | EU016725.1 | EU016783.1 | AF094418.1 | ON685466.1 |
| *Angelica archangelica* | AF110536.1 | U36279.1 | GQ248079.1 | GQ248549.1 | AF094362.1 | OQ064632.1 |
| *Annesorhiza altiscapa* | **ON107487** |  | **ON086534** | AM234812.1 | **ON086697** |  |
| *Anthriscus cerefolium* | MK142826.1 | MK142859.1 | KX676656.1 | MG221255.1 | AF094353.1 | ON685495.1 |
| *Anthriscus sylvestris* | GU395133.1 | KT347806.1 | JN896153.1 | KM360636.1 | FJ385078.1 | AY548228.1 |
| *Aphanopleura capillifolia* |  |  |  |  |  | DQ516368.1 |
| *Aralia spinosa* | AJ431082.1 | U72481.1 | AJ429371.1 | L11166.1 | AF094458.1 |  |
| *Arctopus echinatus* | DQ832351.1 |  | AF271761.1 | AY188414.1 | GQ243952.1 |  |
| *Arracacia aegopodioides* | **ON107408** |  | U58549.2 | U50223.1 |  | GQ862378.1 |
| *Arracacia longipedunculata* |  |  |  |  |  | GQ862406.1 |
| *Arracacia tolucensis* |  |  |  |  | **ON086583** | GQ862429.1 |
| *Asteriscium chilense* | KM672083.1 | **ON107364** | DQ133784.1 | DQ133799.1 | GQ243961.1 | **ON064060** |
| *Astrodaucus orientalis* | AF123748.1 | **ON107346** |  |  | AF094343.1 | FJ415108.1 |
| *Athamanta cretensis* | **ON107437** | **ON107329** | **ON086532** | KF602101.1 | **ON086654** | KT347716.1 |
| *Azorella acaulis* | **ON107492** | **ON107367** |  | AY188423.1 | GQ244007.1 | **ON064067** |
| *Azorella ameghinoi* | KM672061.1 |  |  |  | JX034832.1 |  |
| *Azorella andina* | KM672066.1 |  |  |  | GQ244008.1 |  |
| *Azorella haastii* |  |  |  |  | GQ244023.1 |  |
| *Azorella prolifera* | KM672010.1 |  | AY188402.1 | AY188428.1 | GQ244016.1 | OQ429979.1 |
| *Azorella trifoliolata* | **ON107491** |  | AY188397.1 |  | GQ243976.1 |  |
| *Bifora radians* | **ON107428** | **ON107310** |  |  |  | **ON063969** |
| *Bowlesia lobata* |  |  |  |  | GQ243981.1 |  |
| *Bowlesia tropaeolifolia* | KM672086.1 |  |  |  | GQ243983.1 | **ON064065** |
| *Bupleurum aureum* | KF573906.1 |  | HQ687971.1 | HQ688072.1 |  |  |
| *Bupleurum balansae* | **ON107475** |  | **ON086536** |  | **ON086682** |  |
| *Bupleurum falcatum* | AF110566.1 |  | JQ794929.1 | U50224.1 | **ON086675** |  |
| *Bupleurum fruticosum* | AF110569.1 | **ON107355** |  | D44556.1 | **ON086690** |  |
| *Bupleurum longifolium* |  |  |  |  | **ON086678** |  |
| *Bupleurum praealtum* |  |  | HE970681.1 | HE963363.1 | **ON086683** |  |
| *Bupleurum ranunculoides* | AF110564.1 | U72456.1 |  |  | AF094441.1 |  |
| *Bupleurum rotundifolium* | AF110567.1 | **ON107356** | MK926096.1 | MK925534.1 | AF094442.1 |  |
| *Bupleurum stellatum* | **ON107486** |  |  | HG416974.1 | **ON086688** |  |
| *Bupleurum tenuissimum* | **ON107478** |  | JN895623.1 | JN892975.1 |  |  |
| *Carum buriaticum* | MW589415.1 |  | MN273526.1 | MN204657.1 |  | JQ792206.1 |
| *Carum carvi* | FJ385182.1 | U72435.1 | MN167187.1 | KF602102.1 | AF094392.1 | MT922584.1 |
| *Centella asiatica* | EU188437.1 | U72465.1 | MN886299.1 | D44559.1 | AF094454.1 |  |
| *Chaerophyllum aromaticum* |  |  |  |  | **ON086660** | AM284408.1 |
| *Chaerophyllum aureum* | EF544476.1 | **ON107337** |  | KF602103.1 |  | KJ956488.1 |
| *Chaerophyllum hirsutum* | **ON107440** | **ON107339** |  | KF602104.1 |  | KJ956515.1 |
| *Chaerophyllum temulum* | **ON107441** | **ON107341** | JN895813.1 | KM360710.1 | AF094354.1 | KJ956564.1 |
| *Chamaesium paradoxum* | FJ385184.1 |  | KT280193.1 | KT280087.1 | FJ385085.1 |  |
| *Cicuta virosa* | DQ168974.1 | U72447.1 | LC616754.1 | KF022460.1 | AF094423.1 | OL473016.1 |
| *Conioselinum tataricum* |  |  |  |  | AF094409.1 | (AF008623.1,AF009102.1) |
| *Conopodium majus* | MK142833.1 |  | JN894264.1 | MN447804.1 |  | (KU974043.1,KX165691.1) |
| *Coriandrum sativum* | GQ984010.1 | U36288.1 | ON684596.1 | ON684509.1 | AF094404.1 | ON685496.1 |
| *Crithmum maritimum* | AF110540.1 | U72424.1 | JN894640.1 | KM360739.1 | AF094391.1 | MH298755.1 |
| *Cryptotaenia canadensis* | EF185216.1 |  | HQ593258.1 | HQ590054.1 |  | DQ005964.1 |
| *Cyclorhiza peucedanifolia* | FJ385191.1 |  |  |  | FJ385092.1 | FJ385042.1 |
| *Cymbocarpum erythraeum* | MW166376.1 | MW166364.1 |  |  | **ON086631** | MW166352.1 |
| *Daucus carota* | AF110547.1 | KT347798.1 | JN894953.1 | KM360751.1 | AF094328.1 | X17534.1 |
| *Dendropanax arboreus* | GU055050.1 | U72476.1 | MZ493745.1 | U50244.1 | AF094464.1 |  |
| *Domeykoa oppositifolia* | KM672080.1 | **ON107365** |  |  | GQ243990.1 | **ON064061** |
| *Echinophora chrysantha* | MW166381.1 | MW166374.1 |  |  |  | AF077883.1 |
| *Echinophora tenuifolia* | AF164812.1 |  | **ON086522** |  |  | AH008885.2 |
| *Elaeosticta allioides* |  |  |  |  | **ON086635** | HM229355.1 |
| *Elwendia persica* | MW589431.1 |  |  | KP974250.1 |  | MZ889508.1 |
| *Eremocharis fruticosa* | AF110598.1 | U72463.1 | U58590.1 | U50234.1 | AF094452.1 | - |
| *Eremodaucus lehmannii* | **ON107489** |  |  | MK060096.1 |  | **ON064041** |
| *Erigenia bulbosa* | AF110554.1 |  |  | KF613106.1 | AF094433.1 | (AF008636.1,AF009115.1) |
| *Eryngium campestre* | EU070486.1 | **ON107360** | JN894266.1 | JN892017.1 | MW036660.1 | - |
| *Eryngium ebracteatum* | DQ832360.1 |  | JQ586533.1 | JQ590207.1 | **ON086698** | - |
| *Falcaria vulgaris* | **ON107461** | U72433.1 | **ON086529** | KM360781.1 | AF094396.1 | KC995016.1 |
| *Fatsia japonica* | KF591533.1 | U72474.1 | AB038177.1 | FJ470119.1 | AF094466.1 | - |
| *Ferula glauca* | KJ698390.2 | KJ660498.2 |  |  |  | DQ379406.1 |
| *Ferulago macrosciadea* | **ON107371** | **ON107300** |  |  |  | (-,AJ972904.1) |
| *Griselinia lucida* |  |  | U58628.1 | L11225.2 | GQ244116.1 | - |
| *Gymnophyton flexuosum* | KM672074.1 | **ON107363** |  | DQ133809.1 |  | **ON064057** |
| *Gymnophyton isatidicarpum* | KM672076.1 | **ON107362** |  |  | GQ243997.1 | **ON064058** |
| *Haplosphaera phaea* | FJ385194.1 |  |  | KX527408.1 | FJ385096.1 | MT337432.1 |
| *Harmsiopanax ingens* | JX106153.1 |  |  |  | GQ244054.1 | - |
| *Helianthus annuus* | AJ431093.1 | KU315426.1 | AY215805.1 | AF097517.1 | MZ597518.1 | AF047927.1 |
| *Helosciadium nodiflorum* | EF185223.1 |  | JN895216.1 | KM360639.1 |  | KX513941.1 |
| *Heptapleurum heptaphyllum* | KC952183.1 |  | JN407096.1 | LC692655.1 | KC952271.1 |  |
| *Heracleum pastinacifolium* | AF164801.1 |  |  |  |  | EF043022.1 |
| *Heracleum sphondylium* | AF164800.1 |  | FJ395398.1 | KM360814.1 | AF094369.1 | EF043032.1 |
| *Hermas villosa* | DQ133865.1 |  |  | DQ133810.1 | GQ244002.1 |  |
| *Heteromorpha arborescens* | AF110578.1 | U36294.1 | DQ133790.1 | DQ133811.1 | AF094446.1 |  |
| *Homalocarpus dichotomus* | **ON107493** | **ON107366** | **ON086540** |  | GQ244003.1 | **ON064063** |
| *Hydrocotyle vulgaris* | DQ133870.1 |  | MF159411.1 | DQ133813.1 |  |  |
| *Hymenosporum flavum* |  |  | U58623.1 | U50260.1 | AF094474.1 |  |
| *Klotzschia brasiliensis* | DQ133871.1 |  |  | DQ133814.1 | GQ244009.1 |  |
| *Laserpitium archangelica* | KJ832103.1 | KJ832098.1 |  |  |  | FJ415153.1 |
| *Laserpitium latifolium* | KT347822.1 | KT347754.1 | U58566.1 | KF602108.1 | **ON086548** | **ON063995** |
| *Lecokia cretica* |  | U72450.1 |  |  | AF094432.1 | MZ779125.1 |
| *Levisticum officinale* | GU395138.1 |  | U58567.1 | KU853713.1 |  | KC812809.1 |
| *Lichtensteinia trifida* | EU434669.1 |  |  |  | GQ243932.1 |  |
| *Ligusticum scoticum* | AF123756.1 | U72441.1 | MK925975.1 | KM360852.1 | AF094347.1 | MK694948.1 |
| *Lomatium bicolor* | AF358616.1 |  |  |  |  | KF619629.1 |
| *Lonicera chrysantha* | KC429752.1 | MW242826.1 | MT918125.1 | LC694363.1 | MW242826.1 | EU240676.1 |
| *Meum athamanticum* | **ON107376** | **ON107344** | JN894474.1 | KM360882.1 | **ON086638** | KC676171.1 |
| *Molopospermum peloponnesiacum* | AY838432.1 |  |  |  | **ON086696** | PP794210.1 |
| *Mutellina purpurea* | MW589441.1 | **ON107345** |  |  | **ON086639** | MW587681.1 |
| *Myodocarpus fraxinifolius* | GQ984015.1 |  | AF271747.1 |  | GQ244123.1 |  |
| *Neogoezia gracilipes* | EF185232.1 |  |  |  |  | EF177726.1 |
| *Oenanthe aquatica* | **ON107462** | **ON107347** | JN893874.1 | JN890585.1 |  | AY691926.1 |
| *Opopanax hispidus* | **ON107368** | **ON107297** |  |  | AF094410.1 | **ON063973** |
| *Osmorhiza aristata* | KJ157910.1 | **ON107338** | MK435633.1 | D44578.1 | KJ157774.1 | AY548217.1 |
| *Oxypolis fendleri* | EF185239.1 |  |  |  |  | EF647769.1 |
| *Pachypleurum alpinum* | KJ660441.2 | KJ660460.2 |  |  |  | KJ660840.1 |
| *Panax quinquefolius* | JX896431.1 | KC354697.1 | AB088001.1 | U50250.1 | GQ244072.1 |  |
| *Pastinaca armena* | AF164803.1 | **ON107315** | **ON086518** | **ON086541** | AF094371.1 | MT254215.1 |
| *Pastinaca sativa* | AF110538.1 | U36299.1 | JN895118.1 | KM360919.1 | AF094370.1 | MT254214.1 |
| *Pennantia corymbosa* | GQ984002.1 |  | AJ494844.1 | AJ494842.1 | GQ244126.1 |  |
| *Perideridia kelloggii* | EF185251.1 | U72446.1 |  |  | AF094427.1 | AY246965.1 |
| *Petroselinum crispum* | AF110544.1 |  | AY188405.1 | HM850248.1 | GQ243938.1 | ON685498.1 |
| *Physospermum cornubiense* | AF110556.1 | U72454.1 | MK925872.1 | KF997435.1 | AF094437.1 | AF077904.1 |
| *Pimpinella anisum* | **ON107387** |  | KU499891.1 | KP866817.1 | MW036659.1 | MZ191030.1 |
| *Pimpinella peregrina* | **ON107390** | **ON107325** |  |  | **ON086566** | **ON063933** |
| *Pimpinella saxifraga* | **ON107389** |  | JN894986.1 | KM360934.1 | **ON086564** | MH377864.1 |
| *Platysace ericoides* | DQ133879.1 |  |  | DQ133818.1 |  |  |
| *Platysace lanceolata* | GQ984034.1 |  | GQ983657.1 | AY188434.1 | GQ243892.1 |  |
| *Pleurospermopsis sikkimensis* |  |  |  |  |  | GQ379347.1 |
| *Pleurospermum hookeri* | FJ385233.1 | **ON107340** | HQ824801.1 | **ON086542** | FJ385138.1 | EU236199.1 |
| *Pleurospermum uralense* | AF110560.1 |  | JF955565.1 | JF943473.1 | AF094439.1 | JF977835.1 |
| *Pozoa coriacea* | DQ133880.1 |  |  | DQ133819.1 | GQ244020.1 | MH781242.1 |
| *Prangos ferulacea* | **ON107372** | **ON107299** |  |  |  | KX982519.1 |
| *Pseudopanax crassifolius* | JX106167.1 |  |  | FJ470127.1 | FJ470249.1 |  |
| *Ptilimnium capillaceum* |  |  | KJ773051.1 | KJ773797.1 | **ON086589** | EF647822.1 |
| *Pycnocycla spinosa* | MK397891.1 | **ON107323** |  |  |  | (AY941285.1,-) |
| *Quintinia verdonii* | AJ431077.1 | MK397891.1 | AJ429366.1 | AF299092.1 | MK397891.1 |  |
| *Rutheopsis herbanica* | MT513188.1 | MT513180.1 |  |  | MT513166.1 | MT513155.1 |
| *Sanicula canadensis* | DQ832467.1 | U72461.1 | MF350053.1 | KP643702.1 | AF094447.1 |  |
| *Sanicula crassicaulis* | DQ832379.1 | **ON107358** | MF963663.1 | MF963316.1 |  |  |
| *Sanicula epipactis* | DQ832464.1 | U72460.1 |  |  | AF094448.1 |  |
| *Sanicula europaea* | DQ832468.1 |  | JN894982.1 | MT984543.1 |  |  |
| *Scaligeria napiformis* | EF544474.1 |  |  |  |  | OP985317.1 |
| *Scandix pecten-veneris* | AF123753.1 | U36304.1 | KP900775.1 | KM360973.1 | AF094351.1 | KF234635.1 |
| *Scandix stellata* | MK060089.1 | MK142877.1 |  | MK060101.1 |  | MK050084.1 |
| *Schrenkia vaginata* | **ON107422** |  |  |  |  | KC784697.1 |
| *Seseli annuum* | **ON107400** |  |  | KJ746245.1 | **ON086584** | **ON063960** |
| *Seseli hippomarathrum* | **ON107398** | **ON107304** |  |  | **ON086586** | AY179033.1 |
| *Silaum silaus* | **ON107406** |  | JN894498.1 | JN891403.1 |  | EF560689.1 |
| *Silphiodaucus prutenicus* | MK142839.1 | KT347773.1 |  |  | MK142851.1 | AF336374.2 |
| *Sinolimprichtia alpina* | FJ385251.1 |  | KX526503.1 | KX527312.1 | FJ385158.1 | MT124613.1 |
| *Sium latifolium* | EF185267.1 | U72443.1 | MK926198.1 | KM360984.1 | AF094425.1 | MT108809.1 |
| *Smyrnium olusatrum* | AF110551.1 | U36305.1 | JN895635.1 | MK060102.1 | AF094431.1 | ON685333.1 |
| *Spananthe paniculata* | DQ133887.1 |  | AY188409.1 | U50238.1 | GQ244029.1 |  |
| *Sphallerocarpus gracilis* | **ON107438** | **ON107343** | KX526504.1 | KX527028.1 | **ON086658** | OL472914.1 |
| *Stefanoffia daucoides* | **ON107374** |  |  |  | **ON086607** | (DQ422832.1,-) |
| *Steganotaenia araliacea* | AF110596.1 |  | JF270946.1 | Y10701.1 | GQ243944.1 |  |
| *Thapsia villosa* | KT347839.1 | KT347770.1 | EU531664.1 |  |  | **ON064007** |
| *Thysselinum palustre* | **ON107425** | **ON107305** | **ON086520** |  | AF094384.1 | **ON063944** |
| *Tordylium maximum* | MW166380.1 | MW166372.1 |  | KF997310.1 | **ON086605** | DQ996585.1 |
| *Torilis japonica* | AF123741.1 |  | MN273540.1 | KM361016.1 | FJ385163.1 | MH711118.1 |
| *Torricellia tiliifolia* | AJ431087.1 |  | AJ429375.1 | AF299089.1 | GQ244149.1 |  |
| *Trinia glauca* | **ON107396** |  | MK926110.1 | KF997473.1 | **ON086595** | **ON063947** |
| *Trocdaris verticillatum* | JX974585.1 |  | JN894449.1 | JN891884.1 |  |  |
| *Turgenia latifolia* | AF123743.1 | MK142883.1 |  | MK060104.1 | AF094340.1 | MK050082.1 |
| *Xanthosia rotundifolia* |  |  |  |  | GQ243899.1 | **ON064068** |
| *Zizia aurea* | AF110535.1 | U36307.1 | KT176598.1 | MN601489.1 | AF094367.1 | KF619741.1 |
| *Zosima orientalis* | AF164806.1 |  |  |  | AF094374.1 | AF008628.2 |
